# Rare Etiology of Renal Failure in a 25-Year-Old Caucasian Man: Fabry Disease With a Novel Mutation of GLA Gene

**DOI:** 10.7759/cureus.9136

**Published:** 2020-07-11

**Authors:** Salem Gaballa, Avan AlJaf, Jane Lindsay, Kashyap Patel, Kyaw M Hlaing

**Affiliations:** 1 Internal Medicine, LewisGale Medical Center, Salem, USA

**Keywords:** fabry's disease, painful neuropathy, renal failure, lysosomal storage disease, α-galactosidase a activity, enzyme replacement therapy

## Abstract

Fabry disease (FD) is an X-linked recessive lysosomal storage disease caused by a mutation of the galactosidase alpha (GLA) gene, leading to deficiency of α-galactosidase A (alpha-Gal A). This deficiency results in a progressive, multiorgan accumulation of glycolipids, most notably globotriaosylceramide (Gb3), leading to multiorgan failure and subsequently premature death. Gb3 accumulation in the podocytes, epithelial, and mesangial cells of the glomeruli results in progressive renal disease and eventually renal failure and hemodialysis (HD). There are two types of FD: early-onset classical type 1 and late-onset type 2. Although nearly a thousand mutations of the GLA gene have been identified, the majority of them are of unknown significance. Herein we report the case of a 25-year-old Caucasian male with no significant medical history who presented with peripheral neuropathy and end-stage renal failure, requiring HD. He was diagnosed with FD based on the electron microscopy findings of renal biopsy and severely reduced alpha-Gal A activity (<0.4 nmol/mL/hour). A novel mutation of c.281G>T; p.Cys94Phe was identified. On discharge from our facility, he was referred to a renal transplant center and genetic counseling.

## Introduction

Fabry disease (FD) is the second most prevalent lysosomal storage disorder after Gaucher disease [1]. It is an X-linked inherited mutation of the galactosidase alpha (GLA) gene of the X chromosome [2]. These mutations result in the absence or deficiency of α-galactosidase A (alpha-Gal A) enzyme, which catalyzes the hydrolytic cleavage of the terminal galactose from globotriaosylceramide (Gb3), leading to multiorgan glycosphingolipid accumulations. The prevalence of classic FD is estimated to range from 1:8,454 to 1:117,000 in males, and the disease is seen across all ethnic and racial groups [3]. Diagnosis of FD is challenging; therefore, if physical and clinical examination raises a suspicion of FD, biochemical and/or genetic tests could be considered to confirm the diagnosis [4].

## Case presentation

**Figure 1:**
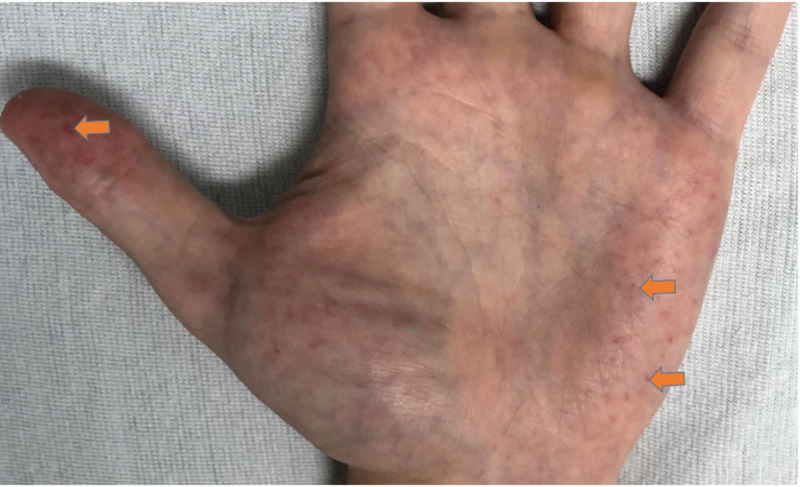
Angiokeratoma of the distal thumb and palm.

A 25-year-old male with no past medical history was brought to the emergency department with complaints of tingling and severe burning sensation in the hands and feet for several days. He endorsed associated nausea and non-bilious emesis, poor appetite, and mental fogginess. He also noted decreased urine output, without any dysuria, hematuria, or lower back pain. He denied any chest pain, palpitation, shortness of breath, abdominal pain, diarrhea, profuse sweating, or heat or cold intolerance. He denied a history of smoking cigarettes or drinking alcohol. He did endorse a family history of FD in his aunt. Physical examination was remarkable for pale conjunctiva, angiokeratoma of fingertips (Figure 1), and asterixis. His vital signs were only remarkable for elevated blood pressure of 180/100.

Complete blood count (CBC) revealed white blood cells of 9.16 cells/mcL (normal range: 4,500-11,000 cells/mcL), hemoglobin (Hgb) of 7.9 g/dL (normal range: 14-16 g/dL), hematocrit (Hct) of 22.6% (normal range for adult males: 40%-50.3%), and platelets of 215 cells/mcL (normal range: 150,000-400,000 cells/mcL). Basic metabolic profile (BMP) revealed sodium of 137 mEq/L (normal range: 135-145 mEq/L), potassium of 4.8 mEq/L (normal range: 3.5-5.2 mEq/L), chloride of 103 mEq/L (normal range: 96-106 mEq/L), carbon dioxide of 20 mEq/L (normal range: 23-29 mEq/L), blood urea nitrogen of 122 mg/dL (normal range: 6-20 mg/dL), creatinine of 21 mg/dL (normal range: 0.8-1.2 mg/dL), glomerular filtration rate (GFR) of 2.7 mL/minute/1.73 m^2^ (normal range: 90-120 mL/minute/1.73 m^2^), calcium of 7.1 mg/dL (normal range: 8.6-10.3 mg/dL), phosphate 9 mg/dL (normal range: 2.5-4.5 mg/dL), and albumin 2.9 of g/dL (normal range: 3.4-5.4 g/dL). Liver function panel was within the normal limits. Troponin was <0.015 ng/mL (normal range: 0-0.015 ng/mL).

Urinalysis showed nephrotic range proteinuria (urine protein/creatinine ratio of 5.07), and microscopic hematuria (>10 red blood cell [RBC], few RBC casts). Erythrocyte sedimentation rate (ESR) was 89 mm/hour (normal range: 0-26 mm/hour). Vitamin B12 was 556 pg/mL (normal range: 254-1,320 pg/mL), vitamin D 25-hydroxy was 26.6 ng/mL (normal range: 30-100 ng/mL), and intact parathyroid hormone was 223.3 pg/mL (normal range: 18.5-88 pg/mL). Iron studies revealed iron of 89 mcg/dL (normal range: 60-170 mcg/dL), total iron binding capacity of 194 mcg/dL (normal range: 240-450 mcg/dL), transferrin saturation of 45.9% (normal range: 20%-50%), and ferritin of 210 ng/mL (normal range: 24-336 ng/mL).

**Figure 2:**
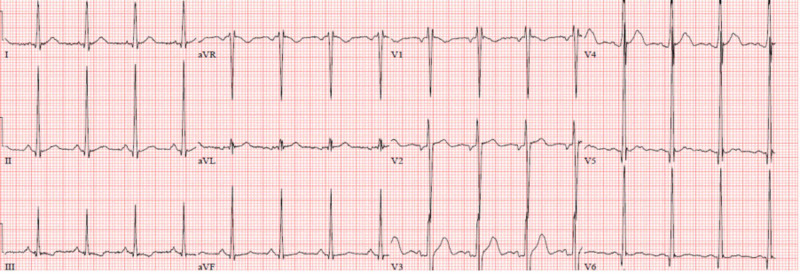
ECG showed normal sinus rhythm, with normal axis and intervals, and LVH. ECG, electrocardiogram; LVH, left ventricular hypertrophy

**Figure 3:**
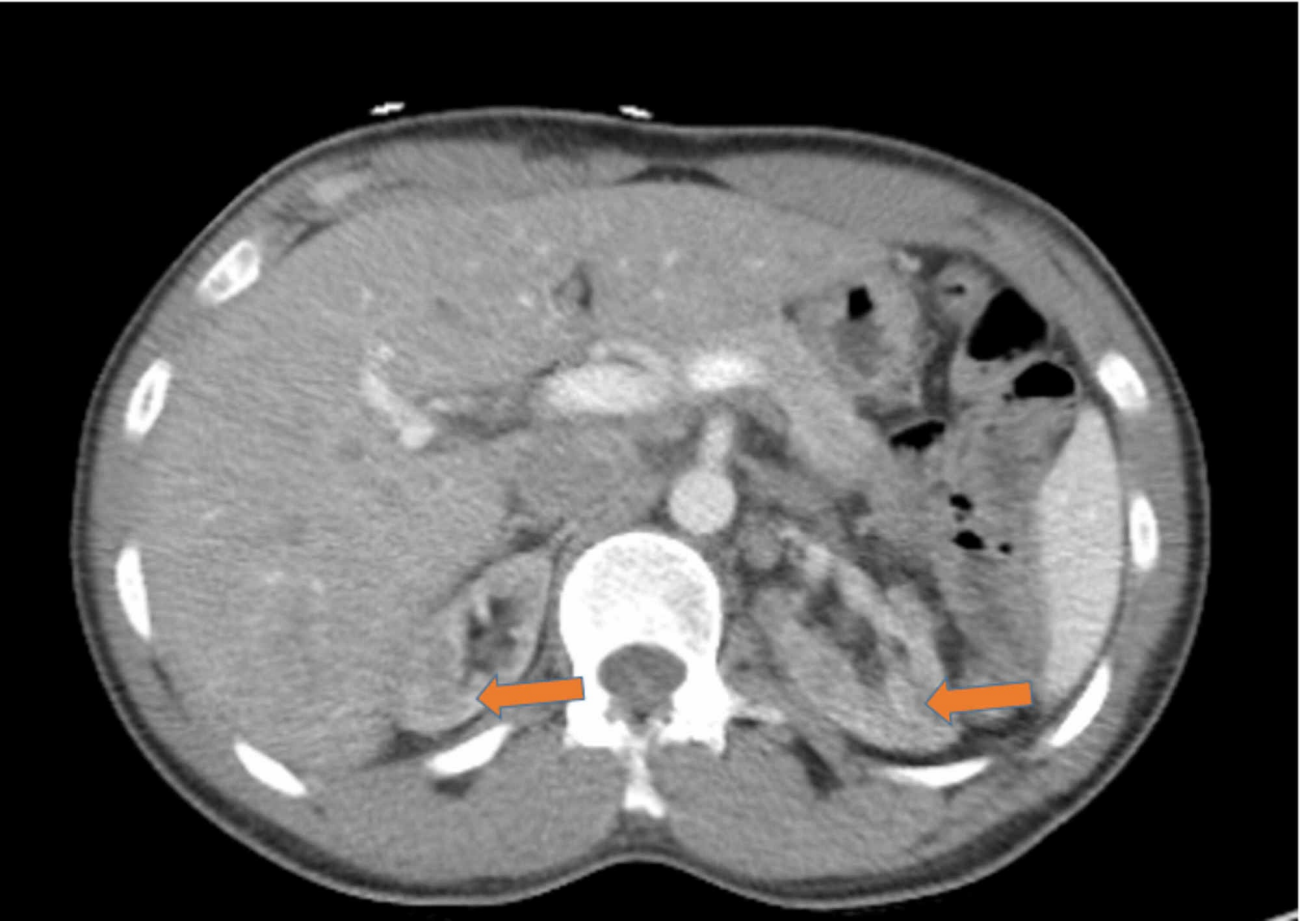
CT of the abdomen and pelvis without intravenous contrast in an axial view showing bilateral renal atrophy, without any evidence of hydronephrosis, pyelonephritis, renal mass, or vascular abnormality. CT, computed tomography

**Figure 4:**
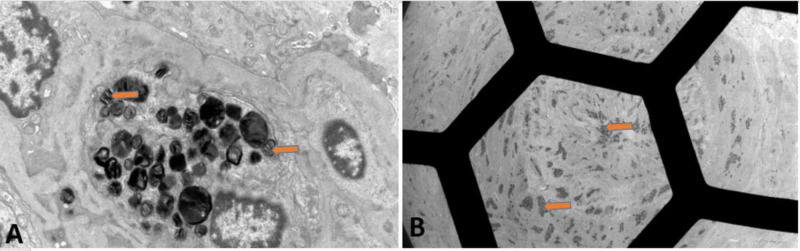
EM of renal biopsy showing (A) numerous electron-dense myelin bodies in the endothelial cell cytoplasm of a glomerular capillary loop and (B) artery with myelin bodies visible within smooth muscle and endothelial cells. EM, electron microscopy

**Figure 5:**
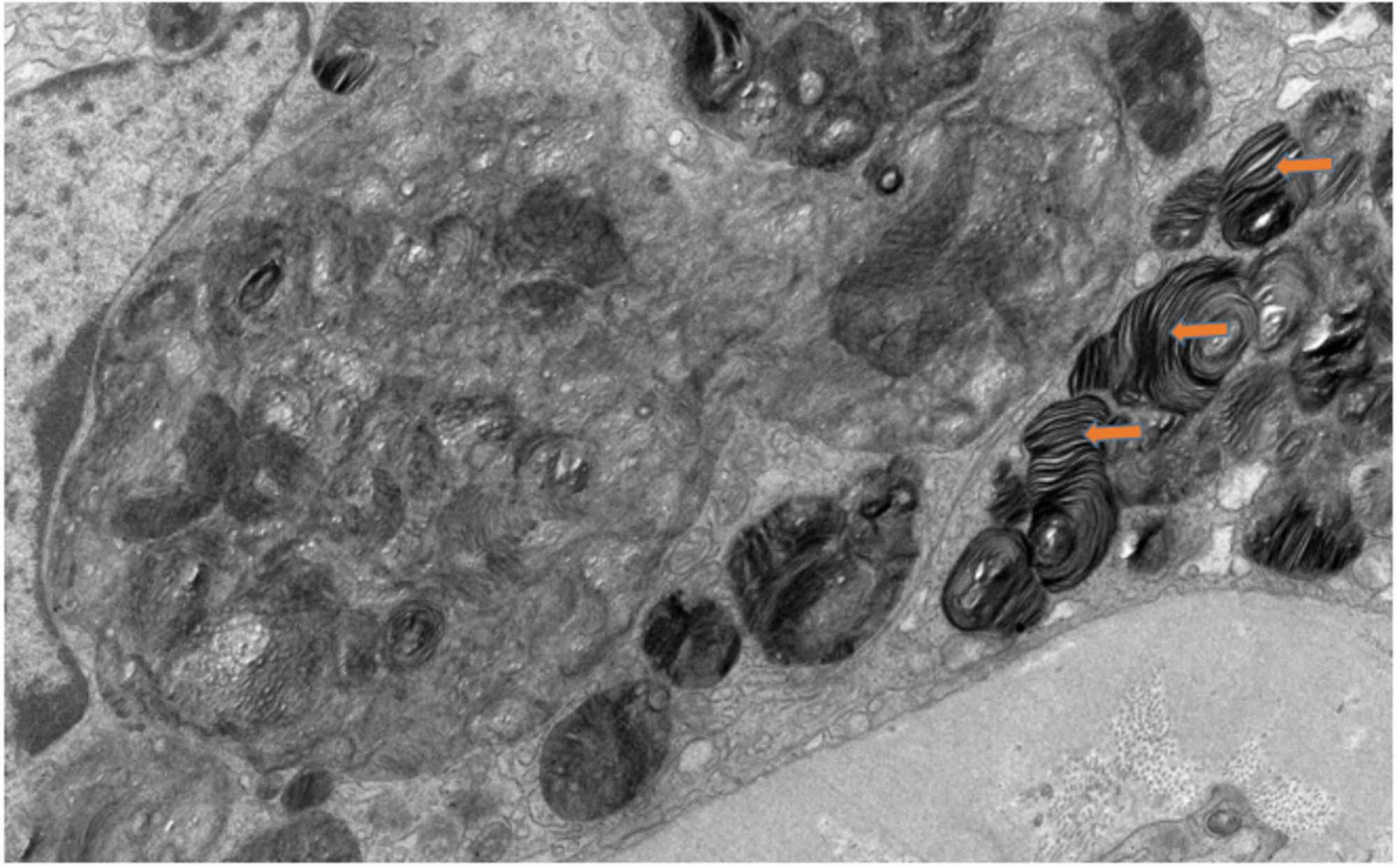
EM showing multilamellated myelin bodies (zebra bodies) within the cytoplasm of a tubular epithelial cell. EM, electron microscopy

**Figure 6:**
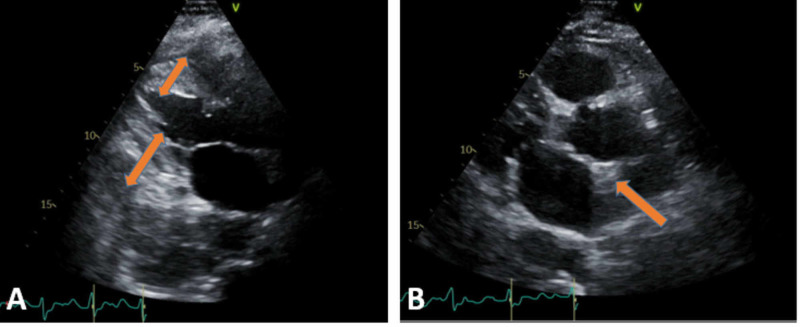
ECHO: (A) TTE in parasternal long-axis view demonstrating concentric LVH and thick IVS, as pointed by the orange arrow, and (B) TTE subcostal view demonstrating concentric LVH. ECHO, echocardiogram; TTE, transthoracic echocardiogram; LVH, left ventricular hypertrophy; IVS, interventricular septum

Electrocardiogram (ECG) showed normal sinus rhythm with left ventricular hypertrophy (LVH) (Figure 2). Computed tomography (CT) of the abdomen and pelvis without intravenous contrast (Figure 3) showed bilateral renal atrophy, without any evidence of hydronephrosis, pyelonephritis, renal mass, or vascular abnormality. Viral hepatitis panel, HIV panel, and toxicology were negative. The antinuclear antibody (ANA) screen, cytoplasmic and perinuclear antineutrophil cytoplasmic antibodies (P-ANCA and C-ANCA), complement levels, and antiglomerular basement membrane (anti-GBM) antibody were all negative. Nephrology service was consulted, and the patient was started on HD due to uremic neuropathy and encephalopathy. Due to the patient’s family history of FD, severe neuropathy, and nephrotic range of proteinuria, the genetic testing, alpha-Gal A activity test, and renal biopsy were performed. The biopsy was limited, with not enough glomeruli for light microscopy (LM) or immunofluorescence microscopy, but electron microscopy (EM) showed numerous electron-dense myelin bodies in the endothelial cell cytoplasm of a glomerular capillary loop, multilamellated myelin bodies (zebra bodies) within the cytoplasm of a tubular epithelial cell, and endothelial cells (Figures 4, 5). Echocardiogram (ECHO) showed mild-to-moderate LVH (Figure 6) and mild pulmonary hypertension with pulmonary artery systolic pressure of 44 mm/hg with an estimated ejection fraction of 55-60%. Alpha-Gal A activity was significantly reduced, <0.4 nmol/hour/mg protein (reference range: 42.1 to 112.9 nmol/hour/mg protein), which confirm the diagnosis of FD. GAL gene sequencing revealed a novel mutation of c.281G>T; p.Cys94Phe.The patient’s peripheral neuropathy and encephalopathy continued to improve on HD, and his blood pressure improved with hydralazine and amlodipine. He was discharged home with continued outpatient HD, with referral to the renal transplant center along with genetic counseling.

## Discussion

The pathophysiology of FD is the absence of significant alpha-Gal A activity that results in the Gb3 accumulation in various cells and tissues, causing cytotoxic, pro-inflammatory, and pro-fibrotic effects [5]. Accumulation of Gb3 is particularly prominent in the vascular endothelium (at levels up to 460-fold higher than normal), vascular smooth muscle cells, and pericytes [6]. The deposition of Gb3 in these cells may lead to the death of smooth muscle cells, vascular occlusion, ischemia, and infarction. There is a relationship between alpha-Gal A enzyme activity and disease symptoms. Mutations that result in little to no alpha-Gal A activity cause the classic early-onset type 1 Fabry phenotype. Those that result in residual alpha-Gal A activity cause the atypical later-onset type 2 phenotype [6]. The prevalence of classic FD is estimated to range from 1:8454 to 1:117,000 in males, and probably underestimated due to the nonspecific manifestations and misdiagnosis of this rare disease [7].

Type 1 FD is the most severe clinical phenotype and occurs predominantly in males, although some heterozygous females have a severe phenotype that resembles classic FD in males [8]. Males with type 1 FD have little or no functional alpha-Gal A enzyme activity (<1% of the normal mean) [8]. Clinical manifestations of classic FD begin in childhood or adolescence and include severe neuropathic or limb pain (acroparesthesias), which occur in more than 75% of patients. Telangiectasias, angiokeratomas, gastrointestinal dysfunction, and corneal opacities (cornea verticillata) are also seen. In adulthood, there are progressive renal manifestations such as proteinuria, or unexplained renal insufficiency, and progressive cardiac and cerebrovascular manifestation such as concentric LVH, heart failure, coronary artery disease, conduction abnormalities, transient ischemic attacks, and ischemic strokes [9]. Other less common and nonspecific manifestations include exercise intolerance, anhidrosis or hyperhidrosis, and hearing loss [9].

Type 2 FD usually presents later in life (third to seventh decades of life) than those with the classic form of the disease. They have residual alpha-Gal A activity (between 2% and 30% of the normal mean) and may not have Gb3 accumulation in capillaries and small blood vessels [10]. Most do not display the classical features of FD, and their disease is typically dominated by a particular organ system, most commonly the heart. The diagnosis is often made incidentally during evaluation of unexplained LVH, heart failure, arrhythmias, proteinuria, kidney failure, or cryptogenic stroke [11].

Renal manifestations are common among males and, to a lesser extent, female patients with FD. Proteinuria, one of the initial renal findings, occurs in approximately 50% of untreated males with classic FD by the age of 35 years [12]. The prevalence of proteinuria in males increases with age, reaching approximately 90% by the age of 50 years [12]. Approximately 30% to 35% of females with FD have overt proteinuria (>300 mg/day), with an onset that is typically later than that in males. A significant fraction of patients develop chronic kidney disease and eventually end-stage renal disease (ESRD) [12]. The prevalence of FD in dialysis populations has been examined in several screening studies. Random screening has identified less than 1% of hemodialysis (HD) patients as having FD. According to Tanaka et al., a screening of alpha-Gal A activity in 696 HD patients (295 females) found only four males and one female (0.7%) to have FD [13].

The pathophysiological changes of Fabry nephropathy are due to Gb3 accumulation in the glomeruli (podocytes, endothelial, mesangial, and parietal epithelial cells), distal tubular cells, and vascular smooth muscle cells. The predilection for podocytes may explain the early renal manifestations of proteinuria. In children with FD, podocyte Gb3 accumulation increased with age and was strongly associated with podocyte foot process width and degree of proteinuria, implicating glomerular involvement and early-onset proteinuria observed in this disease [14]. According to Fall et al., podocyte loss in the urine (podocyturia) is increased in patients with FD and correlates with the clinical severity of kidney disease, suggesting that podocyte loss may be important in the progression of Fabry nephropathy [15]. Kidney biopsy, although not required, may be helpful in establishing the diagnosis of FD. Occasionally, the diagnosis of FD is made incidentally when a kidney biopsy is obtained to evaluate the cause of proteinuria and/or decreased kidney function.

**Figure 7:**
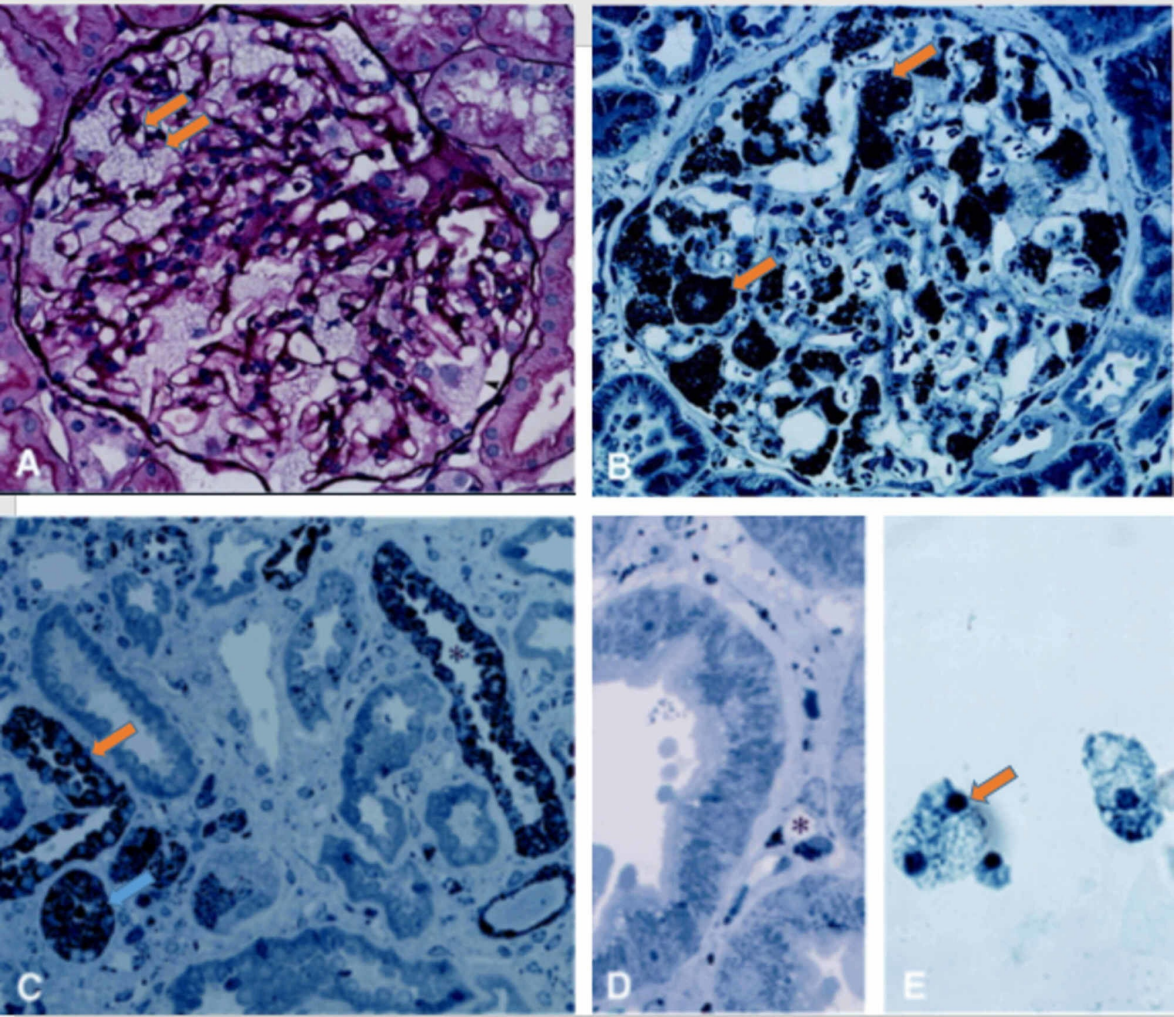
LM of renal biopsy of FD. (A) Glomerulus showing a glycolipids accumulation in the podocytes (arrowhead), with a mesangial widening (PAS stain; magnification, ×80). (B) Embedded renal tissue showing glycolipid accumulation in glomerular podocytes (arrowhead; toluidine blue stain; magnification, ×80). (C) Embedded renal tissue showing glycolipid accumulation in distal tubules (asterisk), with sparing of proximal tubules, and interstitial fibrosis (toluidine blue stain; magnification, ×80). (D) Glycolipid accumulation in the endothelial cells of peritubular capillaries (asterisk; toluidine blue stain; magnification, ×200). (E) Urine demonstrating vacuolated epithelial cells (Papanicolaou stain; magnification, ×160). Figure modified with permission from Branton et al [16]. Image reproduction approved by Wolters Kluwer. LM, light microscopy; FD, Fabry disease

**Figure 8:**
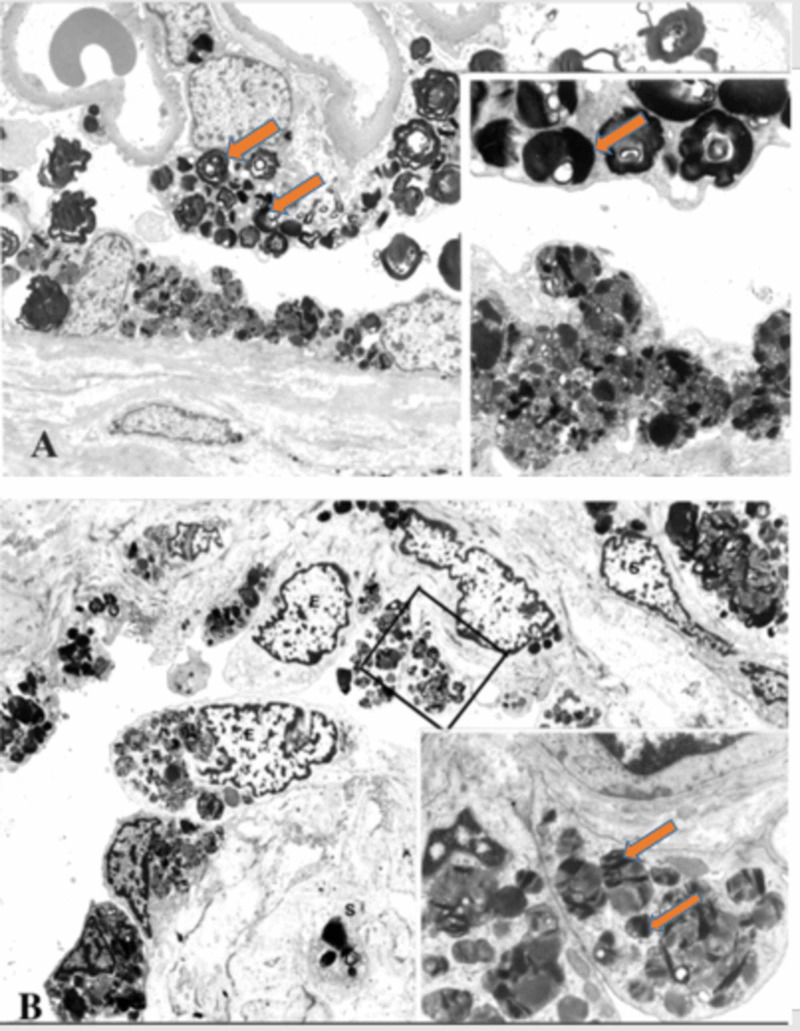
(A) EM of renal biopsy of Fabry disease showing the accumulation of glycolipids within the lysosomes of podocytes and glomerular parietal epithelial cells, with a focal foot process effacement. There is accumulation of glycolipid in the urinary space and a thickened Bowman’s capsule (magnification, ×3760). Higher magnification showing enlarged lysosomes within the podocyte and a combination of multilamellated membrane structures (zebra bodies) and small vesicles in the glomerular parietal epithelium (magnification, ×11,200). (B) EM showing the lysosomal storage within endothelial cells of a small artery and smooth muscle cells (magnification, ×3800). Higher magnification of the endothelial cell cytoplasm showing enlarged secondary lysosomes filled with glycolipids particles of a heterogeneous appearance (magnification, ×11,200). EM, electron microscopy

Abnormal kidney biopsy findings by LM and EM are characteristic in FD. LM shows foamy vacuolization mostly in the visceral glomerular epithelial cells (podocytes) and distal tubular epithelial cells. This is consistent with the described pattern of glycolipid accumulation (Figure 7) [16]. In addition, renal arteries and arterioles show smooth muscle cell Gb3 accumulation as well as smooth muscle degenerative changes representing the death of smooth muscle cells. On EM, deposits of Gb3 appear primarily within enlarged secondary lysosomes as multilamellated membrane structures called zebra bodies (Figure 8) [16].

These inclusions, including concentric layers with a periodicity of 3.5-5 nm and an onion skin appearance, are considered a hallmark of glycolipid storage disorders. Inclusions may be present in all glomerular cell types (most predominantly podocytes), arteriolar smooth muscle cells, and tubules (mostly distal tubules). There is an inverse correlation between the alpha-Gal A activity and renal Gb3 content; thus, glomerular and tubulointerstitial changes and kidney function is worse in patients with undetectable alpha-Gal A activity compared with those with greater than 1% of normal activity [17].

The treatment and prognosis of patients with FD have tremendously changed over the years due to enzyme replacement therapy (ERT), migalastat, HD, and renal transplantation. The main treatment of FD has focused on replacing the missing or deficient enzyme alpha-Gal A with ERT as well as treating the various symptoms and complications [18]. Migalastat is an oral pharmacological chaperone that binds to and stabilizes specific mutant forms of GLA, thereby facilitating proper trafficking of the enzyme to lysosomes and increasing enzyme activity. Therefore, it can be used instead of ERT in patients with amenable genetic variants that allow a substantial increase in enzyme activity. ERT candidates should be evaluated for the presence of certain GLA variants to determine if they may be potential candidates for migalastat therapy. Although ERT may reduce neuropathic pain, its beneficial effects on the severity and/or progression of other disease manifestations are less clear. Hypothetically ERT may reduce tissue accumulation of Gb3 in endothelial cells in the heart, skin, and in most cell types in the kidneys. Therefore, if ERT is used at the early stages of kidney disease, it may slow renal function decline. ERT does not significantly reduce the risk of stroke or heart disease. The short-term and long-term results of kidney transplantation for FD are comparable with transplantation for other causes of ESRD. As with other causes of ESRD, survival with transplantation is superior to that observed with dialysis. Thus, transplantation should be the treatment of choice for ESRD due to FD. A clinically significant Fabry nephropathy does not recur in the transplanted kidney, although some recipients may develop Gb3 deposition that typically does not compromise graft function [19].

Survival is substantially reduced in males with classic FD. Before dialysis, such patients usually died in the fourth decade of life. With the availability of dialysis, life expectancy has increased to the fifth decade [20]. The primary cause of death in patients with FD is from cardiac complications [20].

## Conclusions

FD is an X-linked lysosomal storage disease affecting multiple organs. Clinical manifestations are nonspecific and heterogeneous. The diagnosis is established by a low leukocyte alpha-Gal A activity and a variety of mutations in the GLA gene. LM examination of FD shows foamy vacuolization in glomerular cells, tubular epithelial cells, and vascular cells. EM examination shows myelin or zebra bodies. Although renal biopsy and histopathological findings are not needed for the diagnosis of FD, they are a necessary tool to monitor the renal progression and to evaluate the efficiency of ERT. Patients with ESRD secondary to FD should be referred to renal transplant centers, as renal transplantation improves their prognosis.
